# 1432. Exploring Cell Wall Targets to Overcome *Mycobacterium tuberculosis (Mtb*): Ceftriaxone (CRO) Inhibits Ldt_Mt2_, a Major Peptidoglycan (PG) Synthase

**DOI:** 10.1093/ofid/ofac492.1261

**Published:** 2022-12-15

**Authors:** David C Nguyen, Sarah N Redmond, Khalid M Dousa, Christopher Bethel, Magdalena A Taracila, Qing Li, Sebastian G Kurz, Martin S Pavelka, Krisztina Papp-Wallace, Steven M Holland, Barry N Kreiswirth, Henry Boom, Robert A Bonomo

**Affiliations:** Louis Stokes Cleveland Veterans Affairs Medical Center, Cleveland, Ohio; University Hospitals Cleveland Medical Center, Cleveland, Ohio; Louis Stokes Cleveland VA Medical Center, Cleveland, Ohio; Louis Stokes Cleveland VA Medical Center, Cleveland, Ohio; Case Western Reserve University, Cleveland, Ohio; Case Western Reserve University, Cleveland, Ohio; University Hospitals of Tuebingen, Tuebingen, Baden-Wurttemberg, Germany; University of Rochester Medical Center, Rochester, New York; Case Western Reserve University/ Louis Stokes Cleveland VA Medical Center, Cleveland, Ohio; National Institutes of Health, Bethesda, Maryland; Center for Discovery and Innovation, Hakensack Meridian Health, Nutley, New Jersey; Case Western Reserve University / University Hospitals Cleveland Medical Center, Cleveland, Ohio; Case Western Reserve University/ Louis Stokes Cleveland VA Medical Center, Cleveland, Ohio

## Abstract

**Background:**

Drug-resistant tuberculosis (DR TB) is a deadly, difficult-to-treat infection, and new treatment strategies are needed. Despite the wide success of β-lactams (BLs), DR TB guidelines only include meropenem (MEM) and imipenem (IPM), given with clavulanate (CLA). BlaC, the *Mtb* β-lactamase, hydrolyzes CRO less efficiently than other cephems and β-lactamase inhibitors improve the *in vitro* susceptibility of *Mtb* to CRO. Surprisingly, CRO has not been evaluated in DR TB clinical studies. Moreover, the mechanisms by which CRO disrupts *Mtb* PG synthesis are not well characterized. CRO inhibits Ldt_Mt1­_, but activity against Ldt_Mt2_, an important *Mtb* PG synthase, is unknown. To explore this knowledge gap, we examined CRO inhibition of Ldt_Mt2_. In addition, we investigated if combining CRO with MEM or IPM would lower minimum inhibitory concentrations (MICs) more than each agent alone.

**Methods:**

A panel of *Mtb* isolates was selected for susceptibility testing with a broth microdilution method. Timed electrospray ionization-mass spectrometry (ESI-MS) and inhibition kinetic assays were performed.

**Results:**

CRO MICs ranged 0.25 to 16 µg/mL and lowered ≤ 0.06 to 2 µg/mL with CLA (Table 1). Fractional inhibitory concentration indices for CRO + MEM or IPM was < 0.5 for six isolates, suggesting synergy. ESI-MS captured CRO-Ldt_Mt2­_ acyl-complexes at timepoints 5 to 120 min, and a 158 Da fragment loss was observed; MEM and IPM were unchanged (Table 2, Figure 1). When Ldt_Mt2_ was co-incubated with MEM and CRO together, only MEM complexes were captured. Interestingly, *K_i_*_app_­ with CRO (0.07 ± 0.01 µM) was comparable to that with MEM (0.09 ± 0.01 µM).

**Figure:**
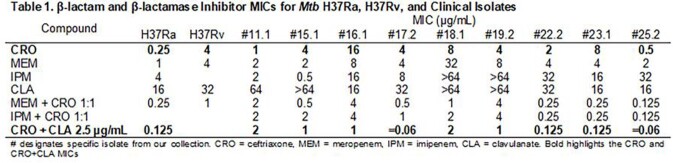

**Figure:**
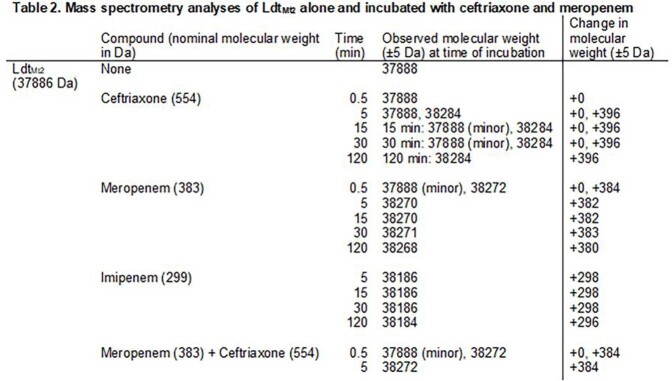

**Figure 1:**
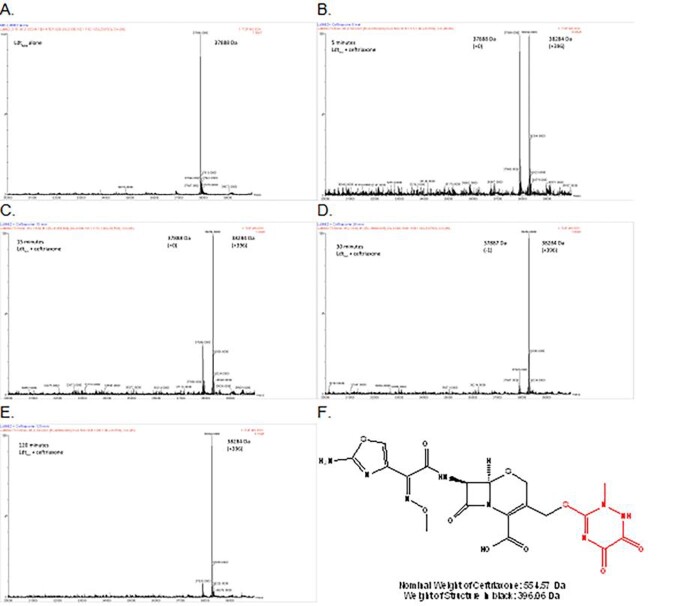
Mass spectrometry chromatograms with LdtMt2 (A) alone incubated with ceftriaxone at (B) 5 min, (C) 15 min, (D) 30 min, (E) 120 min. [Ldt¬Mt2] = 13.2 µM and [ceftriaxone] = 264 µM. Changes in MW are also listed in Table 1. (F) shows the structure of ceftriaxone with the proposed leaving group in red, leaving the remaining bound structure accounting for the observed change in MW.

**Conclusion:**

CRO was effective in lowering MICs with MEM, IPM, and CLA for our *Mtb* isolates. Based upon ESI-MS, we found that CRO forms a stable complex with Ldt_Mt2_ and the R2 side chain is eliminated, while kinetic observations support inhibition of Ldt_Mt2_ by CRO (Figure 2). Previous work found MEM and IPM also inhibit multiple other PG synthases (e.g., PonA1, Ldt_Mt1_, Ldt_Mt3_). We hypothesize that CRO + MEM/IPM inhibits the growth of *Mtb* by the combined inactivation of multiple cell wall enzymes. Our observations support the further exploration of the notion of “target redundancy” as an approach to treat multidrug-resistant mycobacteria with BLs.

**Figure 2.**
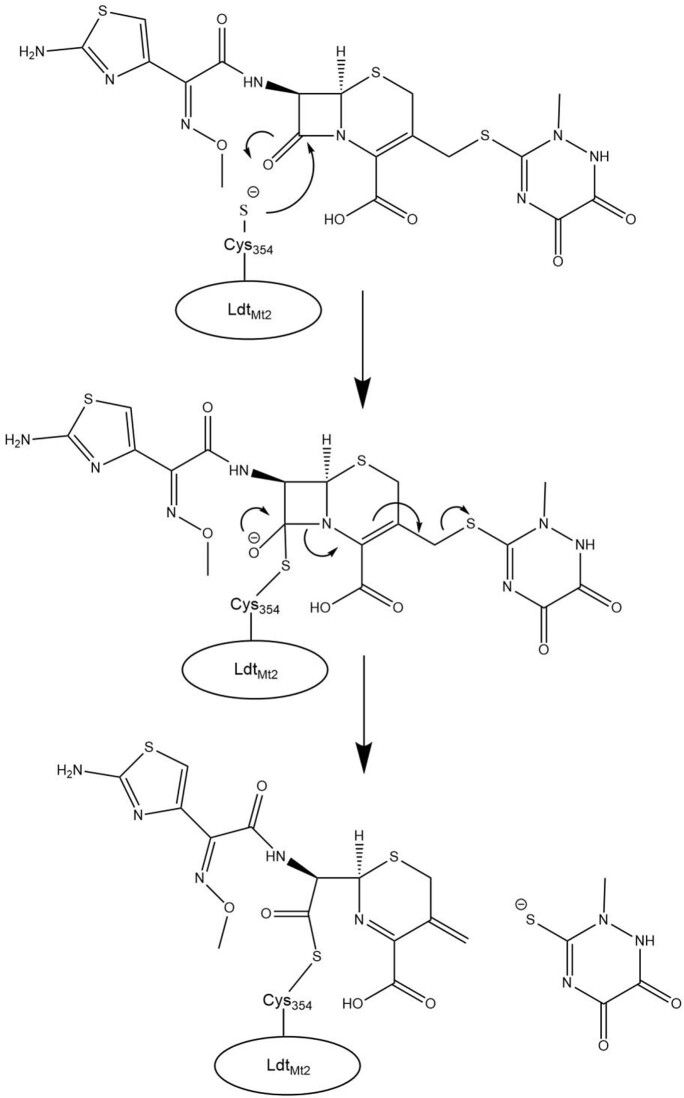
Ceftriaxone-LdtMt2 adduct formation leading to release of the R2 side group

**Disclosures:**

**Krisztina Papp-Wallace, Ph.D**, Merck: Grant/Research Support|Venatorx: Grant/Research Support|Wockhardt: Advisor/Consultant **Robert A. Bonomo, MD**, NIH VA: Grant/Research Support|VenatoRx Merck Wockhardt Cystic Fibrosis Foundation: Grant/Research Support.

